# Platelet lysates in Hepatocellular Carcinoma patients after radiofrequency ablation facilitate tumor proliferation, invasion and vasculogenic mimicry

**DOI:** 10.7150/ijms.44405

**Published:** 2020-07-29

**Authors:** Guoqun Jia, Jian Kong, Changyu Yao, Shilun Wu, Wenbing Sun

**Affiliations:** Department of Hepatobiliary Surgery, Beijing Chao-yang Hospital, Capital Medical University, Beijing, China.

**Keywords:** Platelets, Hepatocellular carcinoma, Radiofrequency ablation, Metastasis, Vasculogenic mimicry

## Abstract

**Background:** Platelets play important roles in tumorigenesis, angiogenesis and metastatic dissemination of tumor cells. Radiofrequency ablation (RFA) could increase the circulating tumor cells in patients with primary or metastatic lung tumors. Whether platelet lysates in hepatocellular carcinoma (HCC) after RFA promote tumor progression has not been elaborated.

**Methods:** HCC patients within Milan Criteria and without taking anti-platelet drugs were selected in the study. MTT assay, colony formation assay, transwell assay, tube formation and western blot were used to evaluate the effect of platelet lysates on HCC cells* in vitro*. Lung metastatic assay was performed *in vivo*.

**Results:** Platelet lysates from patients after RFA promoted cell proliferation, colony formation, migration, invasion and vasculogenic mimicry in Hep3B and HCCLM3 cells compared with those from patients before RFA. Platelet lysates after RFA significantly increased the expression of p-Akt, p-Smad3 and snail, and decreased the expression of E-cadherin compared with those before RFA in Hep3B and HCCLM3 cells. Hep3B-Luc2-tdT cells incubation with platelet lysates from patients after RFA displayed enhanced lung metastasis compared with those before RFA.

**Conclusions:** Platelet lysates from HCC patients after RFA promoted the proliferation, migration, invasion and vasculogenic mimicry of HCC cells, which indicated that RFA in combination with anti-platelet drug may be used to improve the prognosis of HCC.

## Introduction

Hepatocellular carcinoma (HCC) is a major cause of cancer-related mortality worldwide 1. Ablative therapies, in particular, radiofrequency ablation (RFA) are commonly considered a curative option for patients whose tumors are not amenable to resection 2. The recurrence rate of HCC within Milan criteria undergoing RFA is 8-26% in three years 3. Furthermore, tumor progression including rapid growth or metastasis in HCC after RFA could happen.

Platelets play important roles in tumorigenesis, contributing to inflammation, angiogenesis and metastatic dissemination of tumor cells 4. Circulating platelets contain numerous proteins, including growth factors, chemokines and proteases, which are synthesized by megakaryocytes or absorbed from the blood by the platelets themselves 5. Platelets may become activated systemically or within the tumor, potentially resulting in release of platelet content into the circulation 6. Up to 40% of HCC patients have portal vein thrombosis at time of diagnosis, which indicated platelets are activated in HCC 7. The aggregation of blood platelets around cancer cells protect these cells from the immune system and facilitate their circulation in the bloodstream and their adhesion at potential sites of metastasis 8. In addition, tumor-associated platelets can release several permeability factors and enzymes that assist metastatic tumor cells to engraft at distant sites 9.

RFA induced tumor cell dissemination in patients with colorectal liver metastases 10. RFA also increased the circulating tumor cells in patients with primary or metastatic lung tumors 11. Radiofrequency catheter ablation has become standard treatment for the cure and prevention of atrial fibrillation 12. However, thromboembolic events could occur as a complication of the procedure. RFA could indeed favor intra-atrial thrombogenesis through activation of the coagulation cascade related to both catheter placement- and radiofrequency-induced tissue injury. Importantly, platelet activation consequent to atrial endocardial injury likely also plays a significant role in initiating the mechanisms eventually leading to thrombosis 13. However, whether platelets in HCC after RFA promote tumor progression has not been elaborated. In contrast to the heterogeneity displayed by tumor cells, platelets are relatively invariable, and targeting them is potentially, promising method of inhibiting metastasis.

In the present study, we compared the effect of platelet lysates before and after RFA on HCC cell lines. We found that platelet lysates from HCC patients after RFA could promote the proliferation, migration, invasion and vasculogenic mimicry of HCC cells. Furthermore, platelet lysates from patients after RFA accelerated lung metastasis of HCC cells. Combination of RFA and anti-platelet drug may be used to prevent HCC local recurrence and metastasis.

## Materials and Methods

### Patient selection

The study was approved by the ethical committee of Beijing Chao-yang Hospital, the approval number was 2015-科-28, and the need to obtain written informed consent was waived. HCC patients within Milan Criteria and without other severe and serious organ disease, taking anti-platelet drugs and receiving chemotherapy or interventional therapy in our hospital were selected in the study. Milan criteria were defined as one tumor ≤5 cm, or two or three tumors with each tumor ≤3 cm without any vascular invasion or metastasis as observed by computed tomography.

### Blood samples

In each patient, 5 ml blood samples were collected before RFA and 24 hours after RFA. Blood from patients was drawn directly into two plastic tubes containing 3.8% buffered sodium citrate. Samples were spun at 200 g for 15 min at ambient temperature to obtain platelet-rich plasma (PRP). The PRP was transferred to a clean tube and recentrifuged (500 g for 15 min at ambient temperature). The platelet was used to evaluate the effect of platelet lysates on HCC cell* in vitro* and* in vivo*.

### Antibodies

Horseradish peroxidase (HRP)-labeled anti-mouse and anti-rabbit secondary antibodies were from MBL (Nogaya, JPN). Phospho-anti-Akt, p-ERK1/2, p-Smad3, anti-E-cadherin, snail, ERK2, Akt, and Smad3 antibodies were purchased from Cell signaling (Beverly, CA, USA). Anti-β-actin were bought from Abcam (Cambridge, TX, USA).

### Cells

The human HCC cell line Hep3B, Hep3B-Luc2-tdT and HCCLM3 cells were obtained from the National Infrastructure of Cell Line Resource (Beijing, China). Cells were cultured in high-glucose Dulbecco's modified Eagle medium (DMEM) supplement with 10% fetal bovine serum (FBS), 100 U/ml penicillin and 100 μg/ml streptomycin in humidified atmosphere of 5% CO_2_ at 37°C.

### Platelet lysates

Platelets were collected from HCC patients within Milan criteria before and after RFA. The platelets were subjected to several freeze-thaw cycles to disrupt their membranes and release the growth factors stored in the granules in order to acquire the platelet lysates. During the procedure of experiments, the number of platelet used to obtain platelet lysates was 100 times the number of HCC cells. HCC cells were co-cultured with equivalent volume of platelet lysates for the following experiments.

### Cell viability assay

Hep3B or HCCLM3 cells were trypsinzed and seeded into 96-well plates at a density of approximately 3 × 10^3^ cells per well. After 24 h, adherent cells were treated with platelet lysates. After 24 h, 48 h and 72 h incubation, MTT reagent was added to the cells (0.5 mg/ml), and cells were then incubated for 4 h at 37°C. The cells media were removed and 150 μl DMSO were added to each well followed by gentle shaking of the plates to dissolve the formazan crystals. The optical density (OD) was then measured using an automated ELISA plate reader at 570 nm.

### Colony formation assay

Briefly, 6-well dishes were seeded with 1 × 10^3^ viable cells and allowed to grow for 24 h. The cells were then incubated in the presence of platelet lysates in DMEM with 2% FBS for 2 weeks. The colonies obtained were washed with PBS, fixed in 4% paraformaldehyde for 20 min at room temperature and then washed with PBS followed by staining with crystal violet.

### Western blot

HCC cells were collected and cell lysis was performed by using RIPA lysis buffer including protease and phosphatase inhibitors on ice. The extracted protein was quantified by bicinchoninic acid quantification assay. Then, the total cellular proteins were subjected to SDS-PAGE gel and transferred to nitrocellulose membranes. The membranes were blocked with 5% non-fat milk for 1.5 h and then incubated with respective primary antibody overnight at 4°C. Following washing three times with TBST for 10 min, the membranes were incubated with the appropriate HRP-conjugated secondary antibody for 1.5 h at room temperature. The bands were captured with SuperSignal West Pico substrate (Thermo scientific, Rockford, IL, USA).

### Migration and invasion assay

Cell migration assays were operated by a modified Boyden chamber (Costar-Corning, New York, USA). Hep3B or HCCLM3 cells (2 × 10^4^ cells per well) and platelet lysates were added into the upper chamber, and 600 μl DMEM with10% FBS were added into the lower chamber. The chambers were incubated for 24 h. After removing the filter inserts and the cells on the upper side of the filter, the migrated cells on the lower chamber were stained with crystal violet for 20 min, washed with PBS, and captured. Migration was assessed by counting the number of stained cells from 5 random fields.

For cell invasion assay, each insert needed was precoated with Matrigel. The others steps were similar to cell migration assay.

### Tube formation assay

Growth factor-reduced Matrigel (10 mg/ml; BD Biosciences) was thawed overnight at 4°C, and 70 μl was added to each well of a 96-well plate and allowed to solidify for 30 min at 37℃. Hep3B or HCCLM3 cells (2 × 10^4^) were incubated with platelet lysates for 4 h. Capillary tube formation was observed. Total length and number of junctions of the tubes were quantified using ImageJ software and the Angiogenesis Analyzer plugin of the capillary-like structures.

### Lung metastatic assay

Hep3B-Luc2-tdT cells were treated with platelet lysates for 24 h, and cells (1 × 10^6^ cells) were collected, suspended in 100 μl PBS and injected through tail vein. Three weeks after the injection, the mice were sacrificed and the lung tissues were isolated for *in vivo* Imaging System (Carestream Health, Inc.). The number of visible tumors on lung surface was counted.

### Statistical analysis

All values are expressed as the mean ± SD. The data were analyzed using Student's t test or the ANOVA test. A *P* value of <0.05 was considered statistically significant. GraphPad Prism8 (GraphPad Software Inc., San Diego, California, USA) was used for these analyses.

## Results

### Platelet lysates from patients after RFA promote cell proliferation, colony formation, migration and invasion in HCC cells

**Figure 1:**
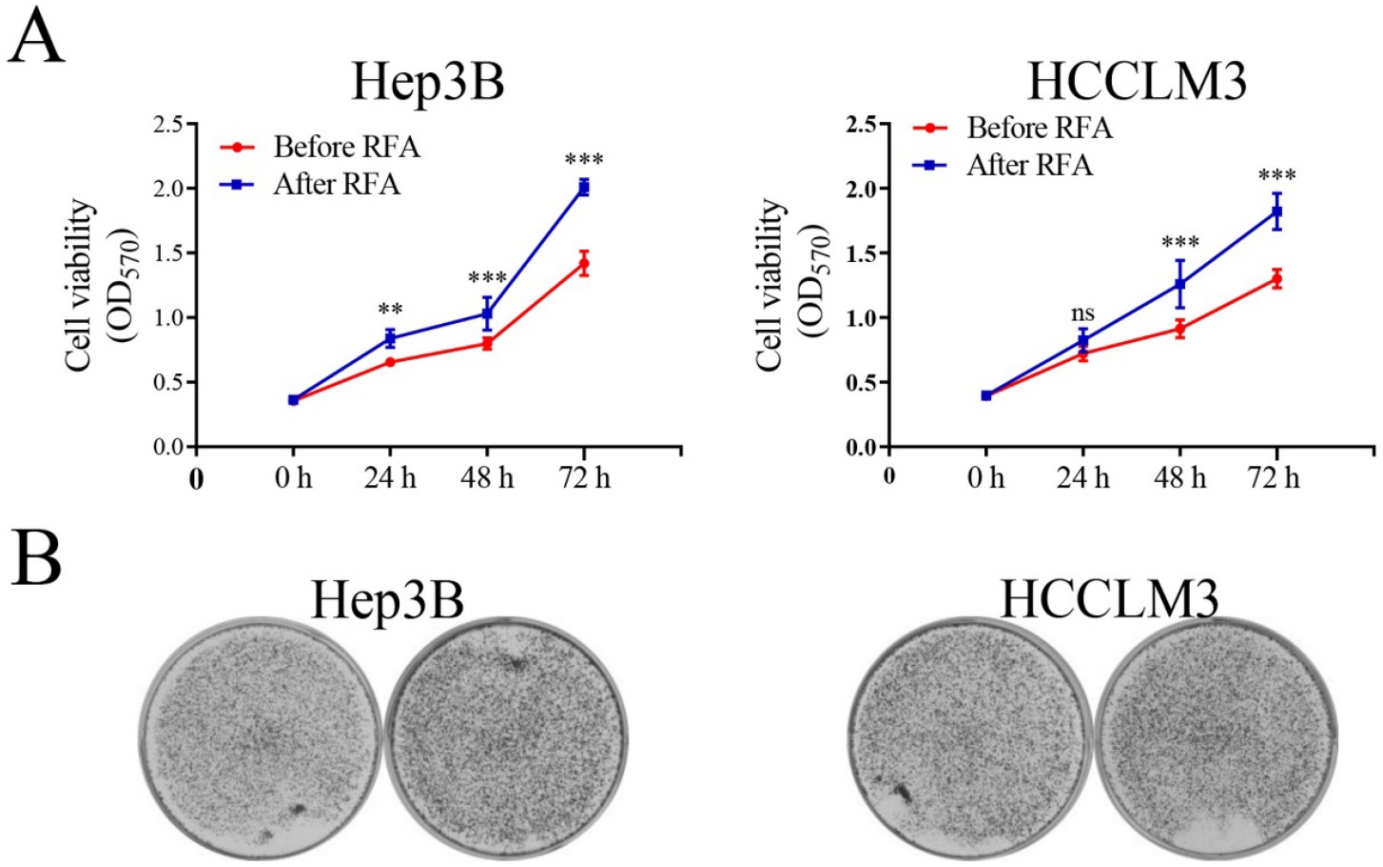
** Platelet lysates from patients after RFA promote cell proliferation and colony formation of HCC cells.** We selected HCC patients within Milan Criteria, acquired the platelet lysates before and after RFA, and examined the effect of platelet lysates on HCC cells proliferation. (**A**) Hep3B and HCCLM3 cells were seeded at 3 × 10^3^ cell per well into a 96-well plate, and incubated with platelet lysates. On 24 h, 48 h and 72 h, MTT was added and OD was measured. (**B**) Hep3B and HCCLM3 were seeded at 1 × 10^3^ cells per well into a 6-well plate, and incubated with platelet lysates. On 14 days, cells were stained with crystal violet. Data are presented as mean ± SD. ns: no significance; ^**^P<0.01;^ ***^P<0.001; ns, no significance.

**Figure 2:**
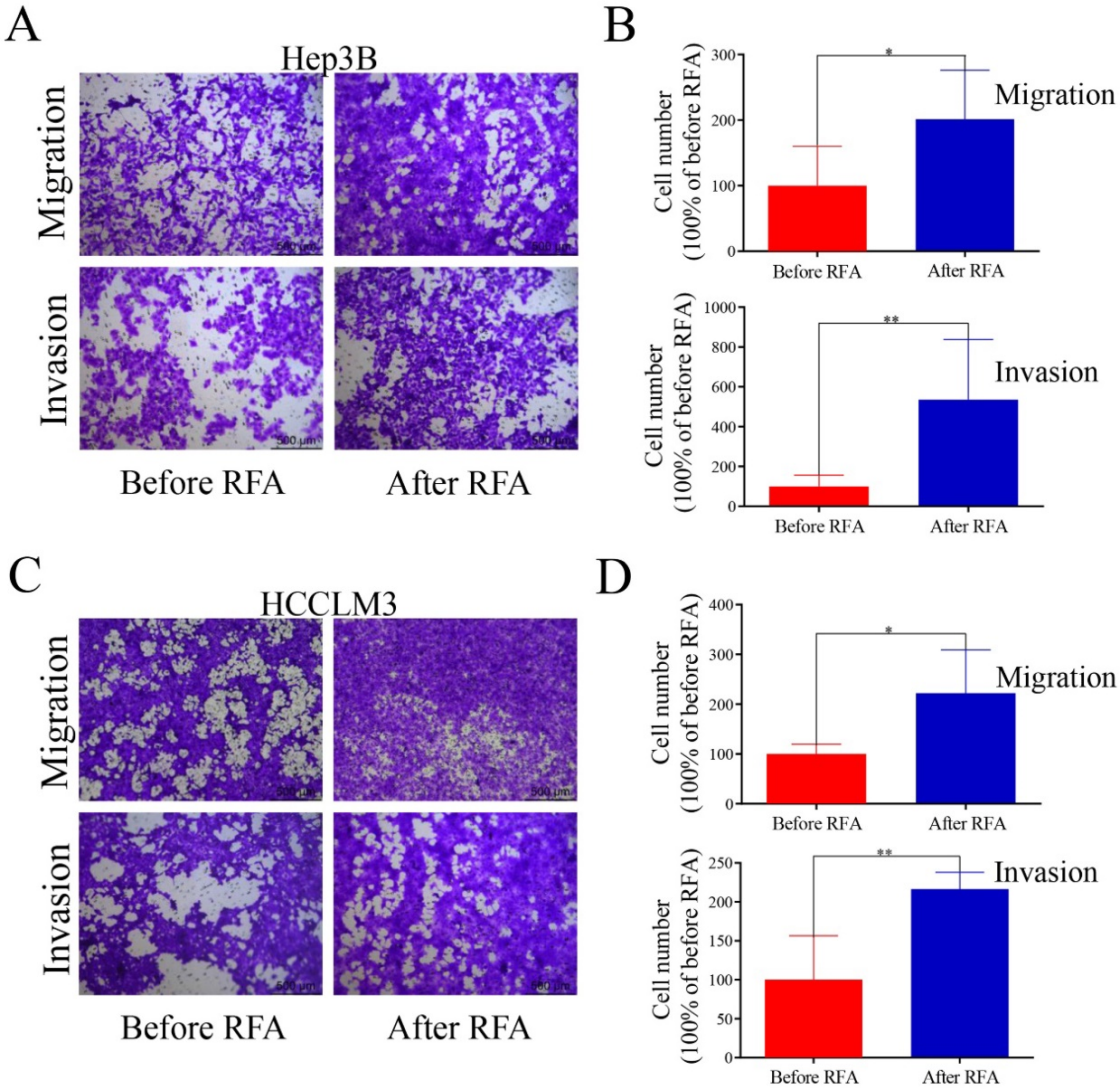
** Platelet lysates from patients after RFA accelerate cell migration and invasion in HCC cells.** Transwell assay was used to examine the effect of platelet lysates on HCC cells migration and invasion. Hep3B and HCCLM3 cells were seeded into the upper chamber, and incubated with platelet lysates. On 24 h or 48 h, migrating or invading tumor cells were examined. (**A, C**) Representative migration and invasion of HCC cells treated with platelet lysates from patients before or after RFA in Hep3B and HCCLM3 cells. (**B, D**) Quantification of tumor cell migration and invasion is shown in Hep3B cells and HCCLM3 cells. Data are presented as mean ± SD. ^*^ P<0.05;^ **^ P<0.01.

We selected patients within Milan Criteria, acquired the platelet lysates before and after RFA, and examined the effect of platelet lysates on HCC cells proliferation, migration and invasion. The characteristic of patients were shown in Table S1. MTT assay and colony formation assay were used to observe the effect of platelet lysates on HCC cell proliferation. Hep3B and HCCLM3 cells after incubation with platelet lysates from patients after RFA displayed increased proliferation and colony formation ability compared with those before RFA (Figure 1). We also evaluate the effect of platelet lysates on HCC cell migration and invasion using transwell assay. Platelet lysates from patients after RFA significantly promoted cell migration and invasion of Hep3B and HCCLM3 cells compared with those before RFA (Figure 2).

### Platelet lysates from patients after RFA facilitate vasculogenic mimicry of HCC cells

**Figure 3:**
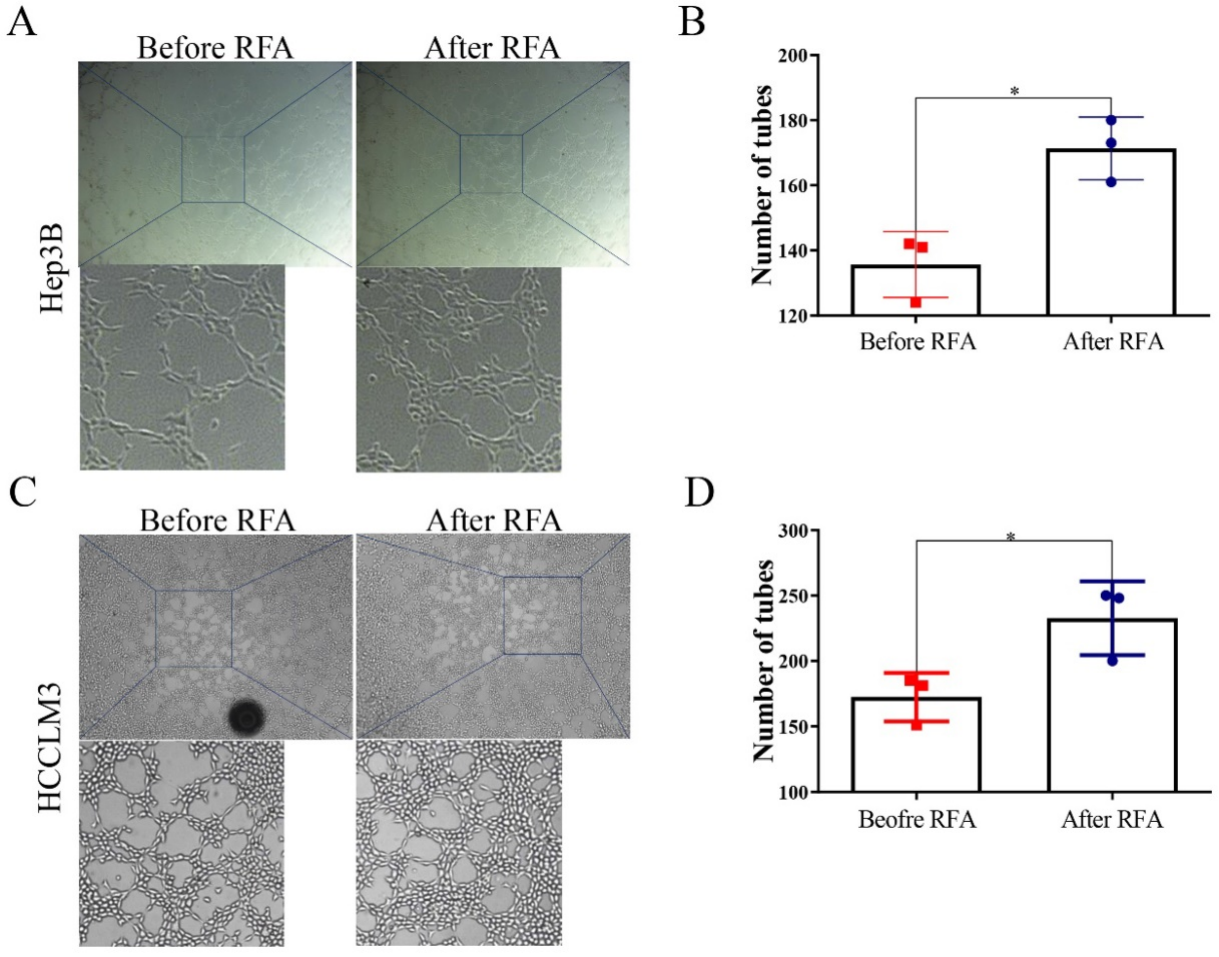
** Platelet lysates from patients after RFA facilitate vasculogenic mimicry of HCC cells.** Tube formation was performed to evaluate the effect of platelet lysates on vascular mimicry in HCC cells. (**A, C**) Representative *in vitro* capillary network formation of HCC cells treated with platelet lysates from patients before or after RFA in Hep3B and HCCLM3 cells. (**B, D**) Quantitative analysis of the mean number of tube-like structures formed using ImageJ in Hep3B and HCCLM3 cells. Data are presented as mean ± SD. ^*^ P<0.05.

To examine the effect of platelet lysates on vasculogenic mimicry, tube formation assay was used. Hep3B and HCCLM3 cells after incubation with platelet lysates from patients after RFA formed more tubes compared with those from before RFA (Figure 3). These data indicated that platelet lysates from patients after RFA were more likely to promote vasculogenic mimicry channels of HCC cells.

### Platelet lysates after RFA may activate epithelial mesenchymal transition of HCC through Akt, ERK1/2 and Smad3 signaling pathways

**Figure 4:**
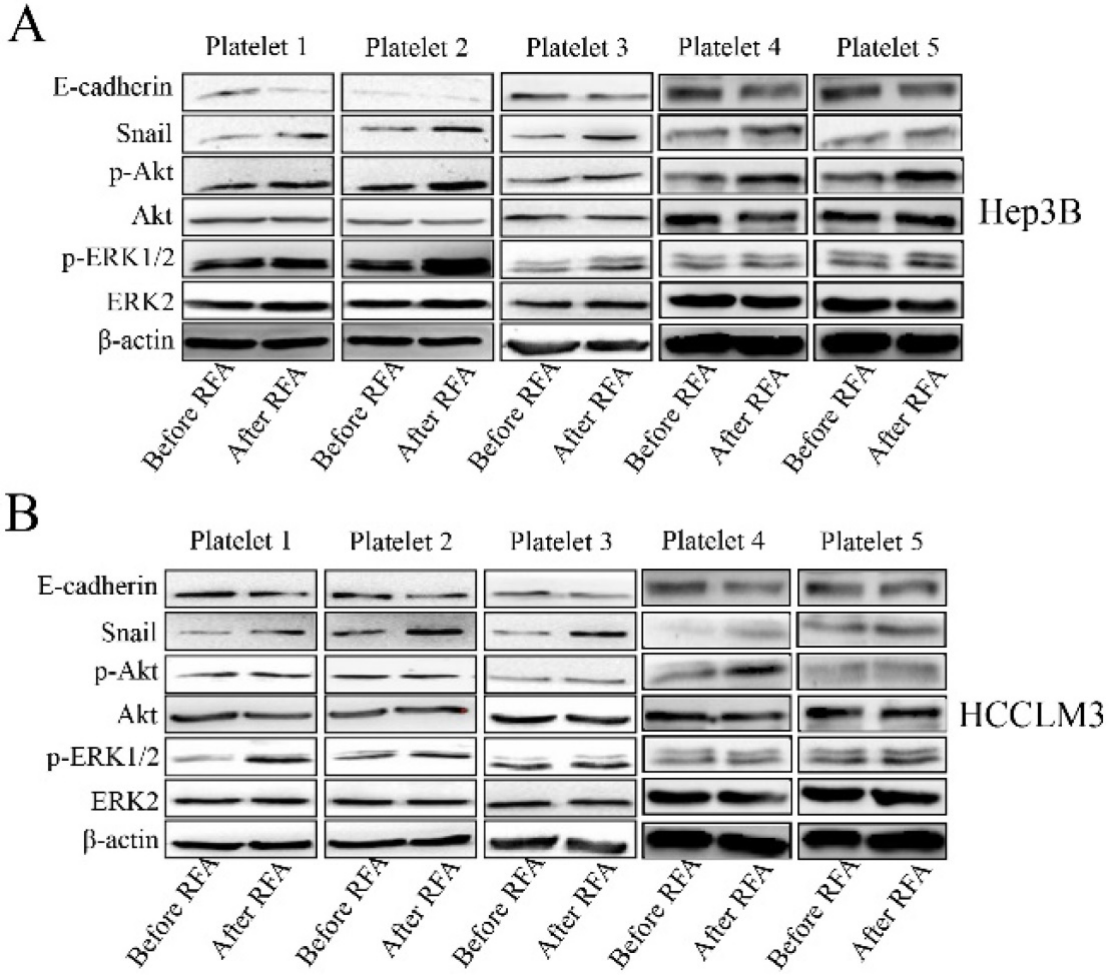

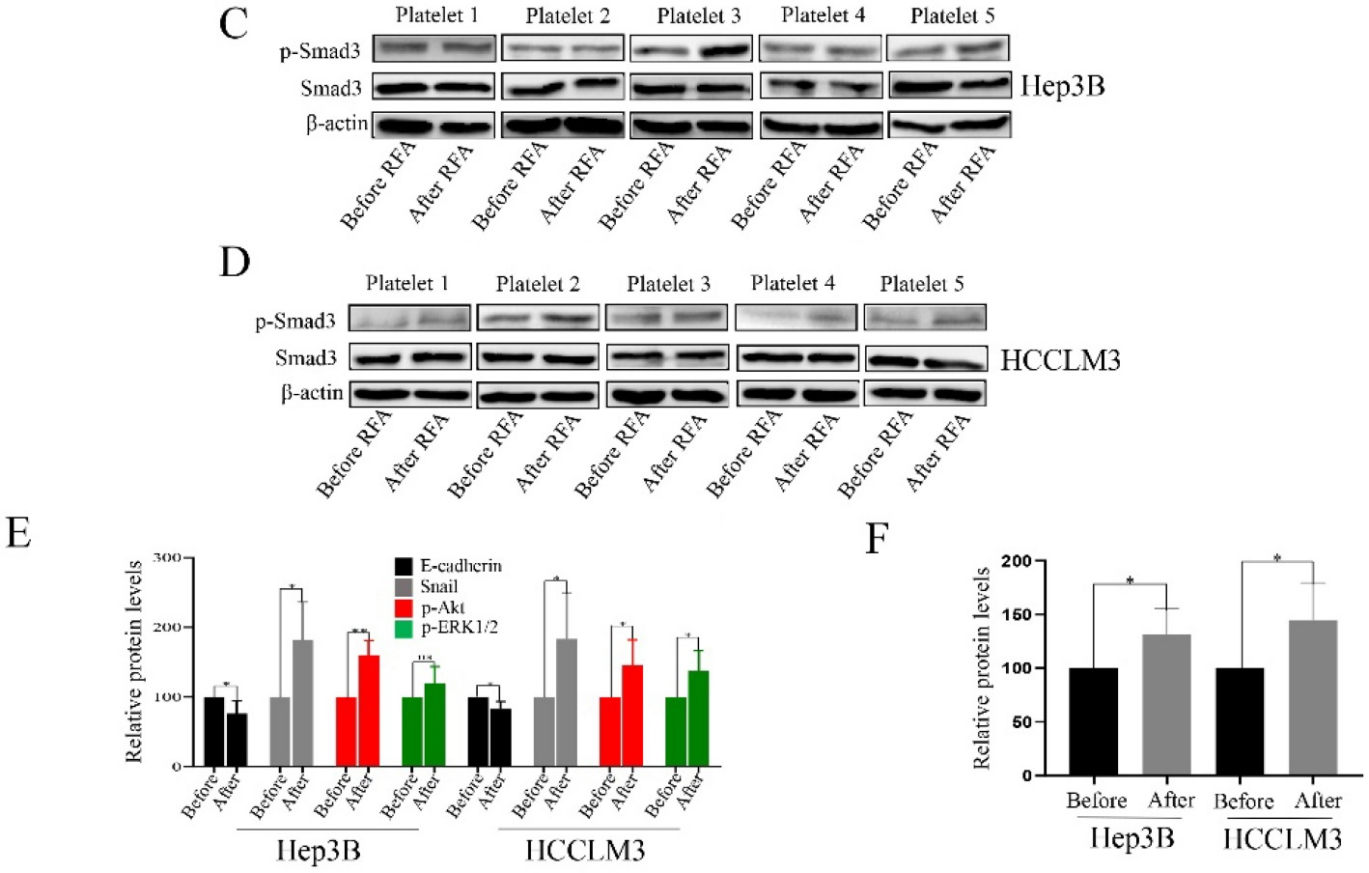
** Platelet lysates activate the Akt, ERK1/2 and Smad3 signaling pathways in HCC cells.** We also investigated the associated mechanism involved in the process. The expression of p-Akt, p-ERK1/2, snail and E-cadherin was examined in Hep3B (**A**) and HCCLM3 cells (**B**) after treatment with platelet lysates from patients before and after RFA using western blot. (**C, D**) The expression of p-Smad3 was examined in Hep3B and HCCLM3 cells. (**E**) The statistical results in the expression of E-cadherin, p-Akt, snail and p-ERK1/2 were shown. (**F**) The statistical results in the expression of p-Smad3 were shown. Data are presented as mean ± SD. ^*^ P<0.05; ^**^ P<0.01; ns, no significance.

We also investigated the associated mechanism involved in the process. We collected 5 pair cases of platelets and observed the effect of platelet lysates. Platelet lysates from patients after RFA significantly increased the expression of p-Akt, and snail, and decreased the expression of E-cadherin compared with those before RFA in Hep3B and HCCLM3 cells (Figure 4 A, B and E). The expression of p-ERK1/2 was upregulated after treatment with platelet lysates after RFA compared with those before RFA in HCCLM3 cells (Figure 4B and E). Platelet lysates form patients after RFA also upregulated the expression of p-Smad3 compared those before RFA in Hep3B and HCCLM3 cells (Figure 4C, D and F).

We further used the ERK1/2 signaling inhibitor (U0126), PI3K signaling inhibitor (LY297002) and TGF-β signaling inhibitor (SB431542) to treat Hep3B and HCCLM3 cells before platelet lysates were added. Platelet lysates after RFA promoted the proliferation, migration, and tube formation of Hep3B and HCCLM3 cells compared with those before RFA, and U0126, LY294002 and SB431542 partly suppressed the effect of platelet lysates after RFA (Supplementary Figure 1-3). Furthermore, pretreated with U0126, LY294002 or TGF-β, Hep3B and HCCLM3 cells still showed faster proliferation treated with platelet lysates after RFA compared with platelet lysates before RFA (Supplementary Figure 1-3). The results showed that ERK1/2, PI3K and TGF-β signaling pathways may be involved in the process of platelet lysates promoting proliferation, migration and vasculogenic mimicry of HCC cells.

### Platelet lysates from patients after RFA promote lung metastasis of HCC cells

**Figure 5:**
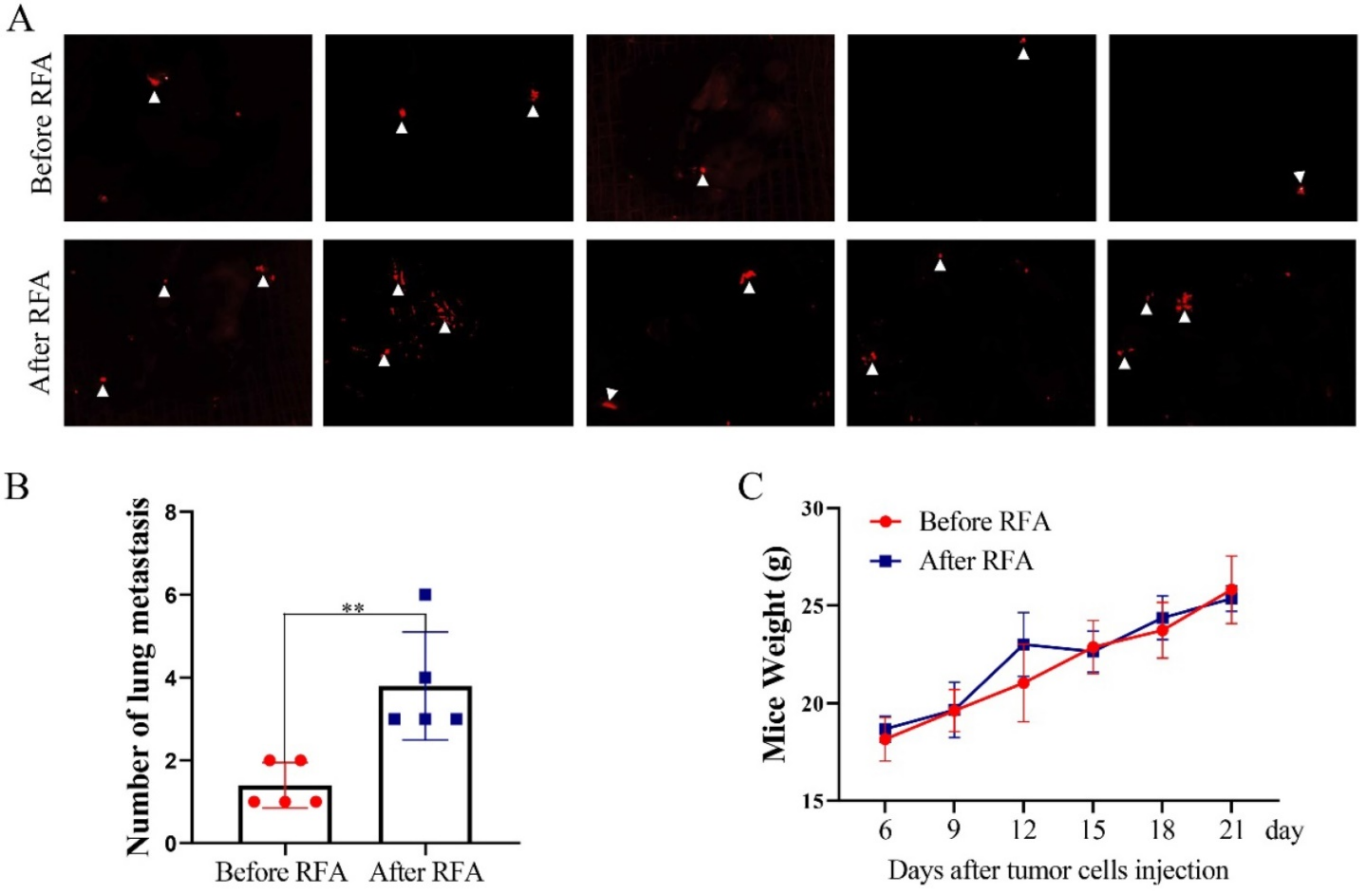
** Platelet lysates from patients after RFA promote tumor lung metastasis.** To evaluate the effect of platelet lysates on tumor metastasis, Hep3B-Luc2-tdT cells after the treatment of platelet lysates from patients before or after RFA were injected through vein tail into mice. (**A**) *In vivo* Imaging System was used to quantify metastatic nodules. Pictures of lung metastatic nodules were shown at the end of the experiment. (**B**) The number of lung metastatic nodules was counted. (**C**) Mice weight was examined every three days. Data are presented as mean ± SD. ^**^P<0.01.

Furthermore, we determined the effects of platelet lysates on the metastasis of HCC cells by *in vivo* Imaging System to quantify metastatic nodules. The number of metastatic nodules was increased in mice injected with Hep3B-Luc2-tdT cells after treatment with platelet lysates from patients after RFA compared with those injected with Hep3B-Luc2-tdT cells after treatment with platelet lysates from patients before RFA (Figure 5A and B). There were no apparent changes in body weight in the two groups (Figure 5C).

## Discussion

Platelets have been recognized as key player in the regulation of tumor metastasis and angiogenesis 14. In the present study, platelet lysates from HCC patients after RFA could promote the proliferation, migration, invasion and vasculogenic mimicry of HCC cells, which indicated that anti-platelet therapy may prevent recurrence and metastasis in HCC after RFA.

Increased platelet activation has been reported in many types of cancer, and activated platelets participate in the progression of the tumor 15-17. Following activation, platelets release extracellular vesicles and granule containing exosomes, microparticles and pro-angiogenic protein, which promote tumor growth, metastasis and angiogenesis. In the field of HCC and platelets most researches focused on the prognostic role of platelet to lymphocyte radio or platelet count in HCC 18. One study demonstrated that no differences in the basal and agonist induced platelets activation status between patients with or without HCC 19. However, another study suggested that anti-platelet therapy could prevent hepatitis B virus-associated HCC 20. Platelets extracts also decreased doxorubicin-mediated growth inhibition and apoptosis induction in HCC cells 21. Platelet extracts induced growth, migration and invasion in HCC *in vitro* 22. Platelet releases promoted the proliferation of HCC cells by suppressing the expression of KLF6 23. In our study, we found that platelet lysates from HCC patients after RFA could promote proliferation, colony formation, migration and invasion *in vitro*, which indicated that anti-platelet therapy may be used to prevent the progression of HCC after RFA.

Vasculogenic mimicry is a novel paradigm of tumor perfusion supported by tumor cells instead of traditional endothelial cells 24. Vasculogenic mimicry functions as a route for metastatic dissemination of tumor cells and is associated with tumor cell migration and invasion, which indicates tumor metastasis and poor prognosis 25. In the present study, we found that platelet lysates from patients after RFA accelerated HCC cell vasculogenic mimicry *in vitro*, which indicated tumor growth and metastasis was enhanced.

Epithelial-mesenchymal transition is involved in the tumor metastasis and vasculogenic mimicry 26, 27. Furthermore, Akt, ERK1/2 and Smad3 signaling pathways participate in the epithelial-mesenchymal transition of HCC 28, 29. Therefore, we observe the effect of platelet lysates on those signaling, and found that platelet lysates from patients after RFA could promote epithelial-mesenchymal transition and Akt, ERK1/2 and Smad3 signaling activation, which suggested platelet lysates may facilitate tumor metastasis and vasculogenic mimicry through Akt/ERK1/2/Smad3 signaling mediating epithelial-mesenchymal transition.

Anti-platelet therapy, especially for aspirin, has shown a promising chemo-preventive agent for many cancer, including colorectal cancer, esophagus cancer, gastric cancer, pancreatic cancer, breast cancer and prostate cancer 30-35. Anti-platelet therapy has also shown protective effects against HCC in preclinical studies and reduces the risk of HCC in chronic hepatitis B 36. Anti-platelet therapy is associated with a better prognosis of patients with hepatitis B virus-related HCC after liver resection 37. Meanwhile, aspirin in combination with TACE could improve overall survival in patients with unresectable HCC 38. The inverse associated between aspirin use and liver cancer was also demonstrated 39. One study demonstrated that aspirin as an anti-inflammation drug could inhibit the rapid progression of residual hepatic VX2 carcinoma following RFA 40. However, in the study, the authors considered that inflammation induced by thermal destruction of the tumor following RFA was the key cause of rapid progression of residual tumor, and aspirin was regarded as an anti-inflammation drug. Our results found that platelet lysates could promote tumor progression in HCC after RFA, therefore aspirin maybe also play the role partly as an anti-platelet drug in the above study. All the results indicated that RFA may combine with anti-platelet drug to prevent the recurrence and metastasis of HCC in the process of RFA.

In conclusion, platelet lysates from HCC patients after RFA promoted the proliferation, migration, invasion and vasculogenic mimicry of HCC cells, which indicated that RFA in combination with anti-platelet drug may be used to improve the prognosis of HCC.

## Supplementary Material

Supplementary figures and tables.
