# Supplementary material for: Platelet lysates in Hepatocellular Carcinoma patients after radiofrequency ablation facilitate tumor proliferation, invasion and vasculogenic mimicry
Source: Int J Med Sci. 2020 Jul 29;17(14):2104–12. doi: 10.7150/ijms.44405 (PMC7484666; doi:10.7150/ijms.44405)

Table S1 The number of red blood cell, white blood cell and platelets in HCC patients before and after RFA

		Patient 1	Patient 2	Patient 3	Patient 4	Patient 5	*P* value
Age (Y)		46	71	63	57	66	
Gender (M/F)		M	M	M	F	M	
RBC	Before RFA	3.7	4.2	4.6	4.1	3.2	0.1216
($\times 10^{12}$/L)	After RFA	3.1	3.9	4.2	4.3	3	
WBC	Before RFA	3.6	4.6	5.9	6.0	3.3	0.1763
($\times 10^{9}$/L)	After RFA	3.1	6.8	8.1	7.2	3.0	
PLT	Before RFA	91	121	116	132	76	0.3171
($\times 10^{9}$/L)	After RFA	82	129	111	125	72	

Supplementary Figure 1. Akt, ERK1/2 or Smad3 signaling pathways inhibition partly suppressed the effect of platelet lysates from patients after RFA on promoting HCC cell proliferation. We used the ERK1/2 signaling inhibitor (U0126, 10 μmol/l), PI3K signaling inhibitor (LY297002, 20 μmol/l) and TGF-β signaling inhibitor (SB431542, 20 μmol/l) to treat Hep3B and HCCLM3 cells before platelet lysates were added. Hep3B cells (A) or HCCLM3 cells (B) were seeded at 3000 cell per well into a 96-well plate, and treated with U0126, LY297002 or SB431542. After 1 h, platelet lysates were added, after 72 h, MTT was added and OD was measured. Data are presented as mean ± SD. $^{*}P < 0.05$; $^{**}P < 0.01$; $^{***}P < 0.001$.

Supplementary Figure 2. Akt, ERK1/2 or Smad3 signaling pathways inhibition partly suppressed the effect of platelet lysates from patients after RFA on promoting HCC cell migration. Transwell assay was used to examine the effect of platelet lysates on HCC cells invasion. Hep3B or HCCLM3 cells (treated with or without U0126, LY297002 or SB431542) were seeded into the upper chamber, and incubated with platelet lysates. After 48 h, invading tumor cells were examined. (A) Representative invasion of HCC cells treated with platelet lysates from patients before or after RFA (with or without the treatment of U0126, LY297002 or SB431542) in Hep3B and HCCLM3 cells. (B) Quantification of tumor cell invasion is shown in Hep3B cells and HCCLM3 cells. Data are presented as mean ± SD. $^{**}P < 0.01$; $^{**}P < 0.001$.

Supplementary Figure 3. Akt, ERK1/2 or Smad3 signaling pathways inhibition partly suppressed the effect of platelet lysates from patients after RFA on facilitating HCC cell vasculogenic mimicry. Tube formation was performed to evaluate the effect of platelet lysates on vascular mimicry in HCC cells. (A, C) Representative *in vitro* capillary network formation of HCC cells treated with platelet lysates from patients before or after RFA (with or without the treatment of U0126, LY297002 or SB431542) in Hep3B and HCCLM3 cells. (B, D) Quantitative analysis of the mean number of tube-like structures formed using ImageJ in Hep3B and HCCLM3 cells. Data are presented as mean ± SD. $^{*}P < 0.05$; $^{**}P < 0.01$; $^{**}P < 0.001$.

Supplementary Figure 1

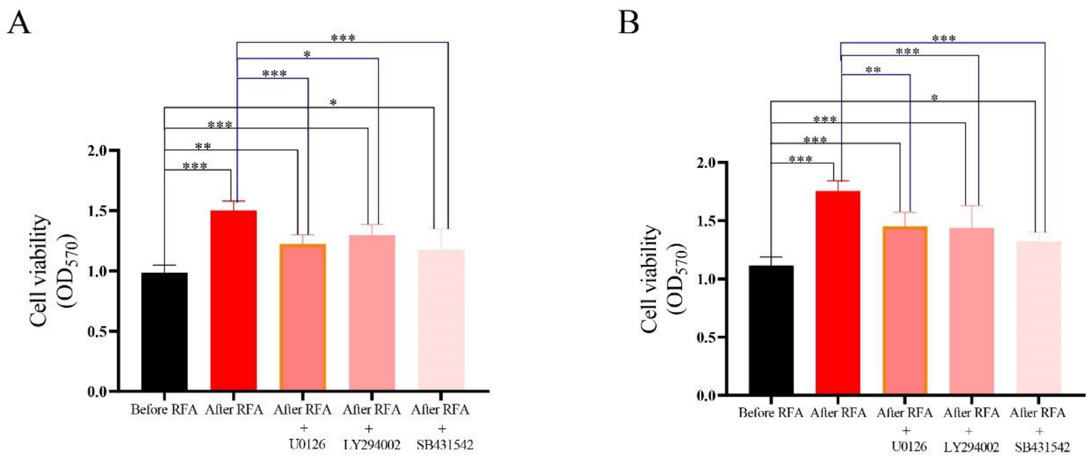

Supplementary Figure 2

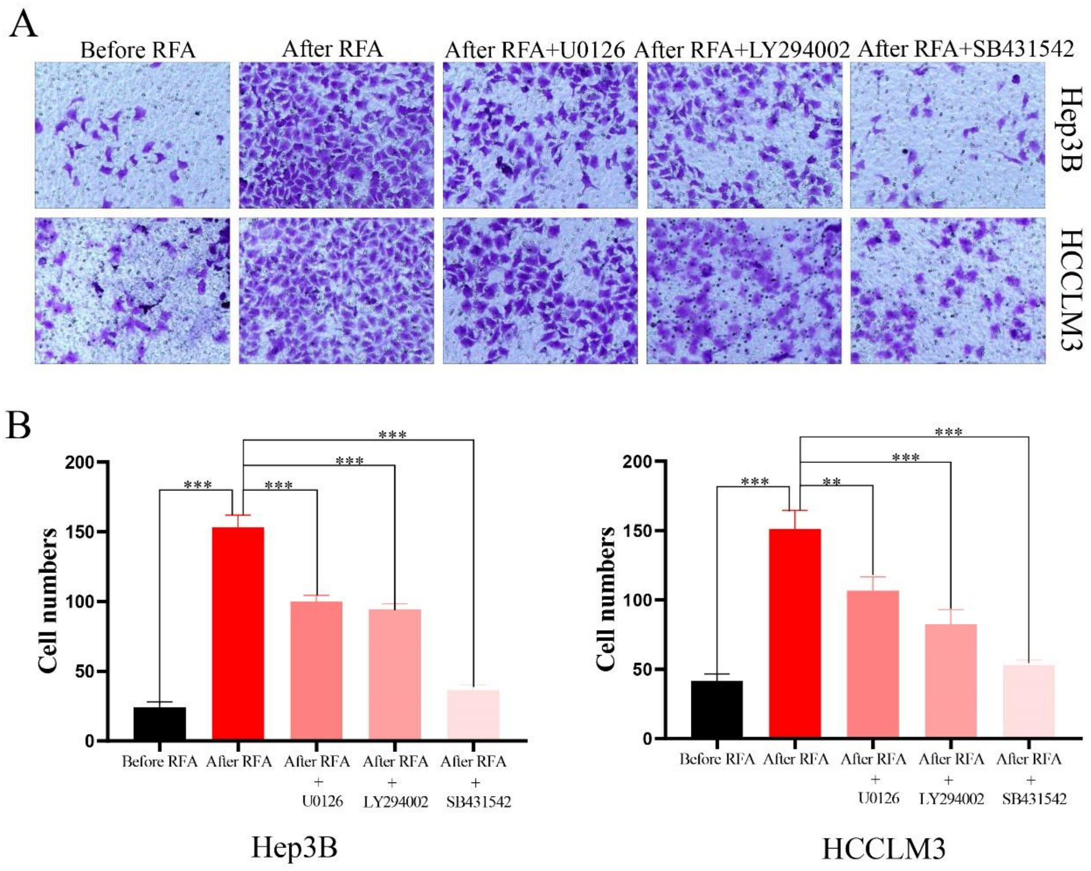

Supplementary Figure 3

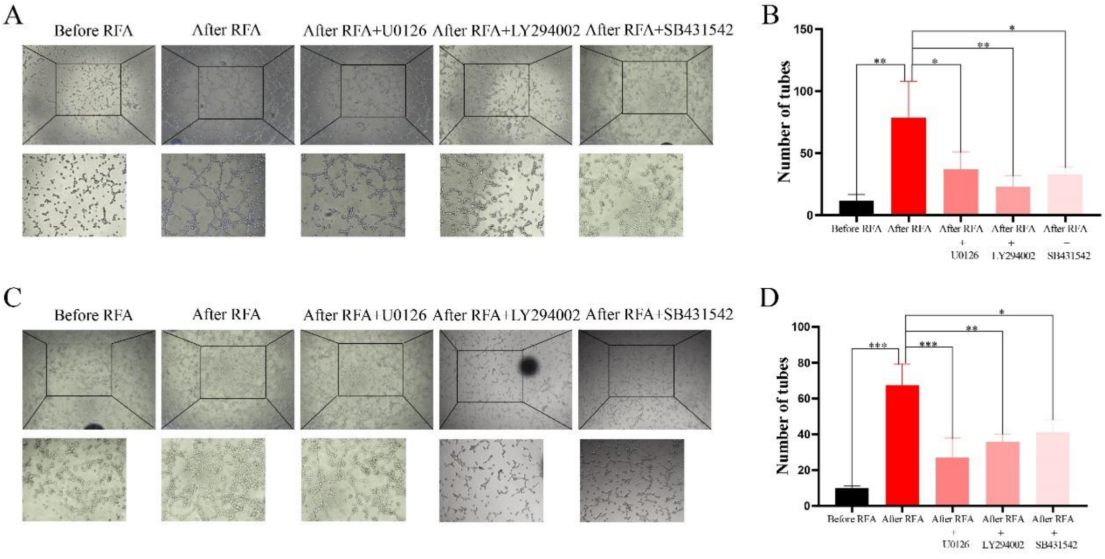

Supplement: Supplementary file 1 — Supplementary figures and tables. [file ijmsv17p2104s1.pdf]
